# Recovery of Phosphate(V) Ions from Water and Wastewater Using Chitosan-Based Sorbents Modified—A Literature Review

**DOI:** 10.3390/ijms241512060

**Published:** 2023-07-27

**Authors:** Łukasz Wujcicki, Joanna Kluczka

**Affiliations:** Department of Inorganic, Analytical Chemistry and Electrochemistry, Faculty of Chemistry, Silesian University of Technology, B. Krzywoustego 6, 44-100 Gliwice, Poland; lukawuj232@student.polsl.pl

**Keywords:** chitosan beads, chitosan hydrogel, chitosan nanofibers, chitosan nanoparticles, orthophosphate(V) removal, orthophosphate(V) adsorption and desorption, phosphorus recovery

## Abstract

Over the past two decades, there has been increasing interest in the use of low-cost and effective sorbents in water treatment. Hybrid chitosan sorbents are potential materials for the adsorptive removal of phosphorus, which occurs in natural waters mainly in the form of orthophosphate(V). Even though there are numerous publications on this topic, the use of such sorbents in industrial water treatment and purification is limited and controversial. However, due to the explosive human population growth, the ever-increasing global demand for food has contributed to the consumption of phosphorus compounds and other biogenic elements (such as nitrogen, potassium, or sodium) in plant cultivation and animal husbandry. Therefore, the recovery and reuse of phosphorus compounds is an important issue to investigate for the development and maintenance of a circular economy. This paper characterizes the problem of the presence of excess phosphorus in water reservoirs and presents methods for the adsorptive removal of phosphate(V) from water matrices using chitosan composites. Additionally, we compare the impact of modifications, structure, and form of chitosan composites on the efficiency of phosphate ion removal and adsorption capacity. The state of knowledge regarding the mechanism of adsorption is detailed, and the results of research on the desorption of phosphates are described.

## 1. Introduction

Phosphorus is an essential element for the proper functioning of living organisms and plants. It is found in various minerals in the environment, mostly as a chemical combination with calcium [1]. Its abundance in the environment indicates that the main source of phosphorus is natural deposits of volcanic and sedimentary origin. It is widely used in agriculture as the main ingredient of fertilizers. Other important uses of phosphorus include the production of detergents, anti-corrosion agents, plastics, and medicines [2].

**Figure 1:**
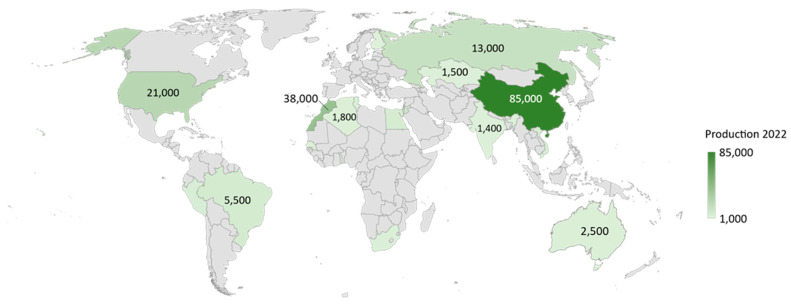
Share of global phosphate mining production in thousands of metric tons (2022 data). Source: own elaboration based on numerical values from reference [3].

The vast applicability of this element led to the increasing degradation of its natural resources, resulting in the balance of phosphorus distribution in the world being altered. Most of the phosphorus used in various anthropogenic activities is obtained from a small part of the ecosystem, where the world’s largest reserves are in China, Morocco, Syria, and Algeria (Figure 1) [3]. This results in dependence and the ability to control the import of phosphorus by these countries. It is estimated that under the current economic and technological climate, phosphorus reserves amount to ca. 71 billion tons and will only last for several hundred years [4]. 

**Figure 2:**
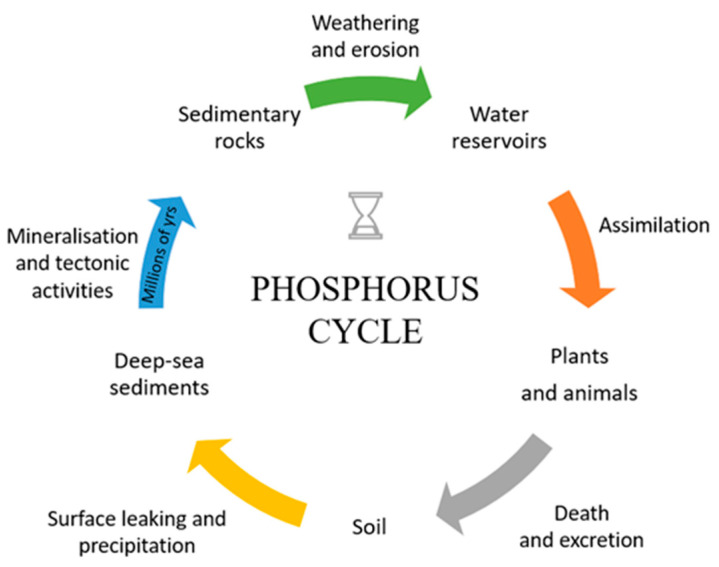
Vector diagram of the phosphorus cycle in the environment. Source: own elaboration.

Economic progress, which contributes to the increased demand for phosphorus, forces specific actions aimed at either replacing such compounds with other substances (e.g., in the case of detergents, substitutes for substances such as potassium pyro- and tripolyphosphates can be aluminosilicates), limiting its amount in some applications (e.g., through the introduction of precision agriculture on a global scale, which will reduce its amounts reaching the soil), or identifying and increasing the share of other sources of its acquisition that would be ecological, cheap, and effective. Unfortunately, the presence of phosphorus in high concentrations has negative effects, e.g., in the aquatic environment, due to the fact that phosphorus is easily transported and tends to accumulate (as shown in Figure 2), and it contributes to the deterioration of water quality in the world through the phenomenon of eutrophication [5,6]. Typically, water bodies contain less than 0.2 mg/L of phosphorus compounds, but areas exposed to agricultural runoff or sewage may contain up to 7 mg/L [6,7]. Therefore, it is crucial to reduce phosphorus levels in water and, if possible, to recover excess phosphorus.

**Figure 3:**
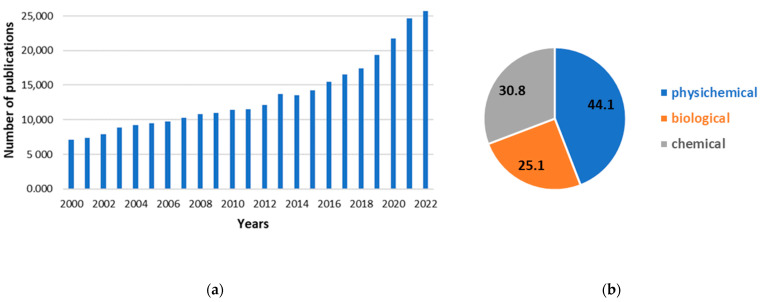
(**a**) The frequency of papers on phosphate removal from the aquatic environment from 2000 to April 2023 (**b**) Percentage of papers on the removal of phosphates from the aquatic environment by biological, chemical, and physicochemical methods. Source: own elaboration based on the Science Direct database.

The significance of the issue of the biogeochemical imbalance of phosphorus in the aquatic ecosystem is evident from the number of yearly scientific publications related to phosphate removal from water and wastewater. In only one Science Direct database, the query “Phosphate removal” received 493,387 responses since 2000, an average of nearly 13,000 annually. Moreover, the number of publications increases every year (Figure 3a).

**Figure 4:**
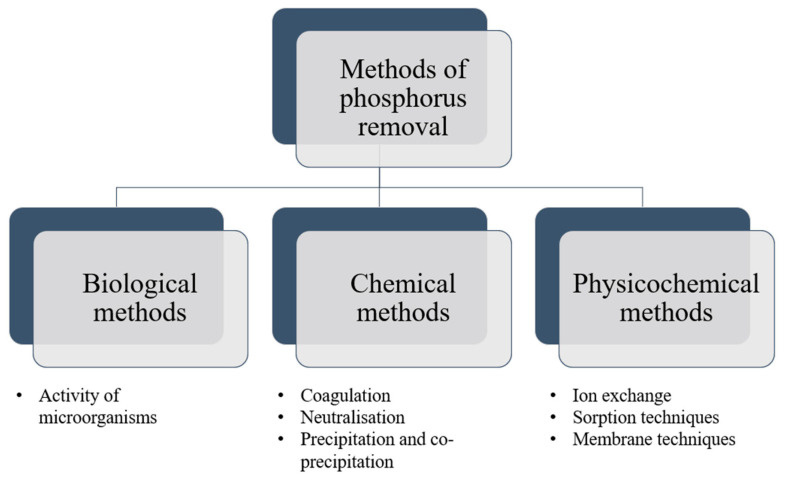
Division of methods for phosphorus removal from aqueous solutions. Source: own elaboration.

**Figure 5:**
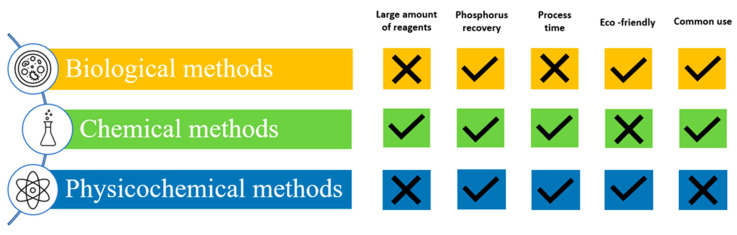
Advantages (√) and disadvantages (X) of phosphorus removal methods. Source: own elaboration.

Due to the interest in the removal of phosphorus from the environment, different ways to remove phosphate from water and wastewater have been developed. Three main categories can be distinguished as follows: biological, chemical, and physicochemical methods, as illustrated in Figure 4. Biological methods involve the utilization of bacteria that can uptake phosphorus, while chemical methods use substances that promote precipitation or coagulation. In contrast, physicochemical methods employ ion exchange, membrane, and adsorption techniques. However, each of these methods has its own set of advantages and disadvantages, as depicted in Figure 5.

Although there are thousands of publications on this topic every year, for the past two decades, as much as 44% of them have focused on physicochemical methods (Figure 3b). In this category, more than half of the articles published in 2022 are concerned with adsorption and ion exchange methods, resulting in almost 17,500 new scientific papers.

Porous materials, such as activated carbon, zeolites, or silica gels, are indispensable materials used as sorbents in the adsorption process to concentrate chemical species on their surface. Biopolymer sorbents, including cellulose, starch, alginate, and chitosan, have become increasingly important since the 21^st^ century. Advances in the preparation and characterization of sorbents have led to the development of pilot installations that use biopolymers in wastewater and water treatment plants. This has provided new opportunities for removing phosphates [8]. One of the valuable biopolymers is chitosan, which has unique properties that make it very advisable to use in some areas, for instance, to remove pollutants from water and wastewater. 

**Figure 6:**
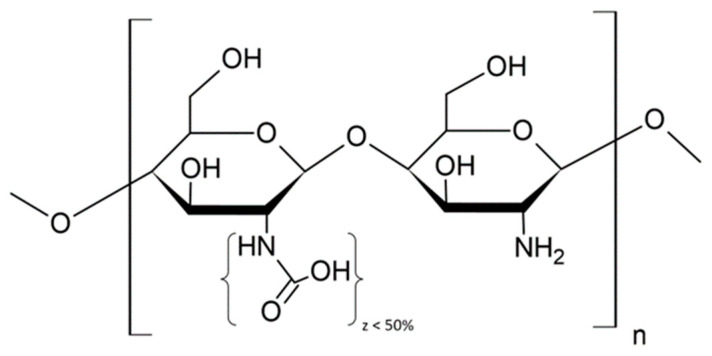
Structural formula of chitosan. Source: own elaboration.

Chitin and its derivative, chitosan, belong to the group of linear polysaccharides. Due to the high prevalence of chitin, which can be obtained from crustacean exoskeletons, insect scales, algae, or fungal cell walls, the availability of chitosan is high. The main difference between chitin and chitosan lies in their solubility and degree of deacetylation. When the degree of deacetylation of chitin becomes higher than 50%, chitosan forms. Chitosan has better properties, which are more commonly used, due to the amount of absence (or lower contribution) of the acetamido groups. Commercially, chitosan is mostly produced by the chemical deacetylation of chitin using parts of crustaceans. Currently, however, because of the increased demand for mushrooms, the acquisition of chitosan from this source is also increasing [9]. Chitosan consists of a random arrangement of residues of d-glucosamine linked by a β-(1,4) bond and *N*-acetyl-d-glucosamine (see Figure 6).

Chitosan belongs to polycationic polymers. Due to the presence of a positively charged amino group in its structure (pK_a_ = 6.5), it increases solubility in acidic and neutral solutions. These properties are primarily related to the structure of the polymer, mainly the molecular weight and the degree of its acetylation, pH, temperature, polymer crystallinity or the form of occurrence (powder, granulate, flake, membrane, etc.), and the presence of functional hydroxyl (-OH) and amino (-NH_2_) groups which can be easily modified by grafting and cross-linking [8,9,10]. Chitosan has a structure that is quite similar to cellulose but with an amino group instead of a hydroxyl group at the C-2 position. This difference has significant implications for the range of applications of chitosan. Chitosan can undergo many reactions and be modified in various ways, making it useful in many different branches of life. Among other biopolymers, chitosan is characterized by its ease of creating various morphological structures, such as films, fibers, hydrogels, nanoparticles, and microspheres. Chitosan has been recognized by the US Food and Drug Administration as a safe compound. This fact points to possible applications of chitosan in various fields, especially biomedicine and agriculture. Other of the most valuable properties of chitosan are biodegradability, bioavailability, antibacterial, and antiseptic, contributing to faster wound healing and adsorption capacity. 

This study focuses on the use of chitosan, in its various forms and modifications, for the removal, separation, and recovery of phosphorus compounds from water systems using the last property mentioned above, adsorption capacity. The following literature review will present the use of different forms of chitosan as a substrate with different fillers and as a composite to remove phosphate(V) from an aqueous matrix from recent literature.

## 2. The Use of Various Forms of Chitosan for the Removal of Phosphates(V)

In this chapter, due to the large number of scientific papers on the discussed issue, it was decided to discuss the achievements in the field of phosphate(V) removal from water and wastewater, dividing the contents according to various forms of chitosan described in the available literature. In addition, only current and innovative methods have been chosen to debate. It was recognized that the adsorption capacity and the degree of phosphorus removal are the parameters through which the application possibilities of the modified chitosan sorbents should be compared. The values of adsorption capacity given by the authors of the referred works were, in most of them, calculated per dry mass of the characterized sorbent. The influence of adsorption–desorption cycles on the efficiency of the process was also added to the discussion if the research results were available.

### 2.1. Chitosan Hydrogels in the Final Form of Wet or Dry Beads

Hydrogel beads are a suitable form of chitosan for the purpose of the adsorption process. The application of chitosan in this form allows convenient separation of sorbent from the solution, as well as contributes to the improvement in the ions’ penetration into the inner sorbent structure, where the adsorption centers are mainly located. One of the fabrication methods of chitosan beads is a coagulation process. This method harnesses the low solubility of chitosan in alkaline solutions, a polymer that precipitates upon contact with an acidic solution with a basic medium. Depending on the droplet-producing method, different sizes of spheres can be obtained: macro-, micro-, or even nano-spheres. Other preparation methods of micro- and nano-spheres include the emulsification of an acidic aqueous chitosan solution in the oil phase combined with a cross-linking and ion gelation of acidic chitosan solution in a solution of tripolyphosphate polyanions [11].

#### 2.1.1. Cross-Linked and Non-Cross-Linked Hydrogel Beads

Unmodified chitosan hydrogel (CSH) has a relatively low affinity for phosphate(V) ions [12]. The low adsorption capacity of CSH occurs at neutral and slightly alkaline pH of the adsorbate, i.e., at the pH value typical for natural waters, for which CSH is used to remove excess amounts of phosphorus compounds. A higher adsorption capacity of CSH is observed at acidic pH (<4), at which uncontrolled and undesirable dissolution of chitosan granules may occur. To prevent the dissolution of chitosan, the physical and mechanical properties of CSH are improved by cross-linking the polymer, but this, in turn, lowers the phosphate adsorption efficiency. 

Chitosan in the form of hydrogel granules (CSHs) without any modification was reported by Jóźwiak et al. [13] at the equimolar mixture of P-PO_4_, N-NO_2,_ and N-NO_3_ to remove anions from aqueous solution. The adsorption capacity of orthophosphates by CSHs reached 15.72 mg/g at pH 4 up to 60 min. 

In another study [14], the efficiency of phosphate removal was compared using non-crosslinked chitosan hydrogel granules (CSHs) and those crosslinked with epichlorohydrin (CSHs-ECH). Two parameters turned out to be crucial for the efficiency of adsorption: the pH of the sorbate and the contact time of the sorbent with the sorbate. It was found that the adsorption of orthophosphates on CSH-ECH was most effective at pH 3; unfortunately, the dissolution of CSH granules occurred under these pH conditions. Considering the stability of CSHs, the optimal pH of phosphate adsorption was set at pH 4. Adsorption capacities of non-crosslinked CSH and crosslinked CSH-ECH were 4.70 mg/g (pH = 4) and 7.86 mg/g (pH = 3), respectively (at the initial phosphate(V) concentration of 10 mg/L P-PO4). In addition, the time of contact of the sorbent with the phosphate solution was very important; it was in the range of 45 to 60 min in the case of orthophosphate adsorption on CSH, while for CSH-ECH it was longer and ranged from 90 to 180 min. Longer contact times than recommended could result in uncontrolled desorption of phosphates into the source solution. In addition, it was found that the desorption efficiency was highest at pH 12–13 and reached values over 90%.

In the Leduc et al. study [15], chitosan hydrogel microspheres (CSHMs) were synthesized by atomization and gelation in sequence, which allowed for the size distribution of microspheres at a micrometric level (average 768 μm) and increased the contact area per unit volume of CSHMs produced. The maximum adsorption capacity was 3.76 mg/g at pH 6.6 and temperature of 293 K, contact time of 30 min, and an initial PO_4_^3−^ concentration of 30 mg/L and CSHMs dosage of 3.34 g/L relative to the dry mass of sorbent. 

Mahaninia and Wilson [16] presented chitosan beads that were cross-linked with glutaraldehyde (GA) and epichlorohydrin (EP), and a systematic adsorption study of phosphate dianion (HPO_4_^2−^) species was carried out in aqueous solution at pH 8.5 and 295 K. It was stated that the favorable adsorption of phosphate dianion was equal to 52.1 mg/g and the elution process using 0.05 M NaCl (aq.) with moderate ionic strength was efficient (desorption efficiency of 95%).

#### 2.1.2. Hydrogel Beads Modified with Metal Ions or Metal Oxides

The use of metal ions for the adsorptive removal of phosphates by chitosan hydrogels increases the adsorption capacity. This may be related to the strong interaction between the positively charged metal cations and negatively charged orthophosphate anions and can be explained by various theories such as electrostatic attractions, Pearson’s theory of hard and soft acids and bases, or ion exchange [12,13,14,15,16,17]. Metals that promote a significant increase in phosphate adsorption include polyvalent metals, i.e., La^3+^, Al^3+^, Zr^4+^, and Ce^3+^. Modification with these metals enhances the adsorption capacity of the phosphates above 100 mg/g of the sorbent [18,19].

Iron ions can be utilized to modify chitosan hydrogel. Karthikeyan et al. [17] studied phosphate removal onto metal (Fe^3+^) loaded chitosan and alginate biopolymeric hybrid beads (Fe–CS–Alg) from an aqueous solution. The obtained adsorption capacity of the synthesized Fe–CS–Alg beads towards phosphate was 84.74 mg/g, which was excellent compared with other reported materials. Adsorption saturation was reached after 40 min, and the favorable pH was 3–6. The regeneration experiment was performed using 0.1 M NaOH solution as a desorbing agent, where the adsorption capacity only moderately decreased with increasing regeneration cycle. 

Particularly, the sorbent modified with lanthanum ions displayed one of the highest phosphate adsorption capacities compared to other modifications. High adsorption is related to the strong affinity of lanthanum(III) to phosphorus(V). Zhang et al. compared the adsorption capacity of phosphates using modified chitosan sorbents filled with lanthanum, zirconium, and iron hydroxides [18]. The highest adsorption capacity (q_m_ = 160 mg/g) was obtained for hydrogel immobilized with La(OH)_3_ nanoparticles within quaternary–aminated chitosan (CS–La–N–20%), also in the presence of salt ions. The pH results indicated that CS–La–N–20% effectively sequestrated phosphate over a wide pH range between 3 and 7 without significant La^3+^ leaching. CS–La–N–20% maintains 84% of the Initial adsorption capacity after seven consecutive adsorption/desorption recycles with NaOH–NaCl desorption solution and continuous stirring at 60 °C. 

In another study, Zhao et al. used La^3+^ ions to modify the chitosan hydrogel but with a protective coating of polydopamine (PDA), which improved the hardness and mechanical strength, and the resultant La–CS@PDA sorbent still exhibited distinct selectivity for phosphate [19]. In addition, the multifunctionality of PDA (associated with the presence of hydroxyl and amino groups in the polymer structure) increased the number of active sites and, thus, a higher adsorption capacity to 195 mg/g. After saturated adsorption (24 h), La–CS@PDA beads were eluted with the mixed solution of 0.3 M NaCl and 0.1 M NaOH for 3 h to achieve almost complete desorption of 96%. 

Another report [20] synthesized lanthanum-modified sludge biochar chitosan (La–SBC–CS) microsphere by dropping sludge biochar (BC) and chitosan into a lanthanum chloride solution. A maximum adsorption capacity of 81.54 mg/g for phosphates at 25 °C was found. The desorbing agent was 3 M NaOH solution with a constant temperature oscillation for 24 h, and the phosphate desorption after one cycle was equal to ca. 94%. Furthermore, La–SBC–CS maintained 76.37% phosphate removal efficiency after eight cycles.

In addition to the high adsorption capacities of lanthanum-based chitosan hydrogels, sorbents modified with zirconium ions also show a high affinity for phosphorus compounds. For example, Liu and Zhang [21] studied the adsorption of phosphates on a chitosan sorbent modified with Zr^4+^ ions (ZCSB). Fourier transform infrared spectroscopy analysis (FTIR) confirmed the cross-linking reaction between zirconium and chitosan and the active adsorption of phosphates (q_m_ = 62 mg/g). The regeneration and reuse of ZCSB after the batch phosphate adsorption experiments were conducted through immersion in 0.5 M NaOH solution as a de-phosphate agent. After the adsorption/desorption fifth cycle, the actual phosphate adsorption efficiency was even retained at 92%. 

Another study that employed zirconium was by Chen et al. [22]. The authors synthesized a chitosan sorbent modified with polyethylene polyamine and Zr^4+^ ions and showed a high degree of phosphate removal (q_m_ = 104 mg/g) and the possibility of using the presented sorbent in sewage reclamation. The use of polyethylene polyamine increased the amount of -NH_2_ groups, which also increased the adsorption capacity of the phosphates.

Zr(IV)-cross-linked carboxymethyl cellulose/carboxymethyl chitosan hydrogel (Zr–CMC/CMCS) was prepared using polyethylene glycol (PEG) as a pore-forming agent and applied for phosphate adsorption. The maximum adsorption amounts of 93.5 mg/g were observed at pH 2.0 for Zr–CMC/CMCS; however, the efficiency dramatically decreased when pH increased. The reusability study suggested that the hydrogel exhibited relatively stable adsorption capacity after six adsorption–desorption cycles with 0.8% (*w*/*v*) NaOH solution and under vibration at 25 °C [23].

In another paper [24], hydrogel beads composed of only zirconia and chitosan (HZCSB) were fabricated using a recipe free of both cross-linker and filling substances and used for phosphate removal. Careful selection of a suitable Zr/chitosan ratio (i.e., zirconium/amine molar ratio of 1:1) resulted in HZCSB possessing good phosphate adsorption performance and good mechanical and chemical stability for the purpose of column systems. HZCSB had a higher adsorption capacity (42.02 mg/g) at pH 6.7 and a similar adsorption rate when compared with Ferrolox, a commercial filtering material for phosphate removal (adsorption capacity of 25.58 mg/g). The desorption efficiency exceeded 95% at 0.5 M NaOH concentration.

Among other polyvalent metals used to modify chitosan hydrogels, cerium ions are worth mentioning. Cerium has a chelating ability with the functional groups present in chitosan and possesses a high adsorption capacity towards anions [25]. Hu et al. researched the adsorption removal of phosphate by Ce(III)-impregnated cross-linked chitosan complex (Ce–CCS), obtaining a maximum adsorption capacity of 45 mg/g at pH 3 [26]. The presence of co-anions decreased the adsorption of phosphate onto Ce–CCS; however, Ce–CCS showed a higher adsorption capacity than cross-linked chitosan (CC) due to the introduction of Ce(III). Furthermore, Ce–CCS was prone to regeneration with 0.1 M HCl solution. In another study, a chitosan-β-cyclodextrin sorbent with embedded cerium Ce^3+^ ions (Ce–C–β–CD) was prepared [27], which promoted the adsorption capacity of the multilayer equal to 89 mg/g. Regeneration and reusability experiments were conducted for up to seven cycles with 0.1 M of NaOH as a desorbing agent to assess the reusability of the prepared microspheres. The obtained Ce–CS–β–CD sorbent in the form of microspheres was tested in practical conditions for the removal of toxic anions from industrial wastewater.

Ma et al. [28] showed a total recycling strategy of selective phosphate removal from wastewater. A novel granule chitosan inlaid with γ-AlOOH on its structure (γ-AlOOH@CS) was prepared, which exhibited a high adsorption capacity of 45.82 mg/g with stability and favorable selectivity at pH 4–6, fast adsorption rate in bath experiments, and performed well in the column experiment for wastewater treatment. After recycling the sorbent using NaOH solution as a solvent and phosphoric acid as a precipitant under hydrothermal reaction conditions, regenerated chitosan for new γ-AlOOH@CS preparation was obtained, and AlPO_4_ was recovered and used in the production process.

There are known modifications of the chitosan hydrogel beads that employ less valuable metals, i.e., Zn^2+^, Cu^2+^, or Ca^2+^. However, the sorbents created from these cations are, in general, not characterized by high adsorption capacities compared to sorbents modified with metals with higher positive charges.

Chitosan beads carboxylated cross-linked with glutaraldehyde loaded with Zn(II) (ZnCCSB) were reported by Sowmya and Meenakshi [29] for the removal of phosphate. The maximum adsorption capacity of ZnCCSB was 67.50 mg/g in the range of pH 4–7. The successful regeneration of ZnCCSB was carried out using 0.025 M NaCl for 30 min of contact time.

Yazdani et al. [30] described three different bio-sorbents developed from CS and zinc compounds; Zn(II)–CS, ZnO–CS, and nano-ZnO–CS in the dry form were investigated for phosphate removal. It was stated that the introduction of Zn(II) ions into chitosan improved its performance towards phosphate uptake from 1.45 to 6.55 mg/g (the initial concentration of phosphate was 5 mg/L) and provided fundamental information for developing bio-based materials in water remediation.

Sorbents based on chitosan beads (CSB) and copper ions (Cu(II)) modified with/without a traditional cross-linking agent (glutaraldehyde) were prepared and were referred to as CSB–G–Cu and CSB–Cu, respectively [31]. The maximum phosphate uptake for CSB–Cu was 53.6 mg/g within 12 h to achieve optimal phosphate removal at pH 7.

Kumar and Viswanathan [32] developed novel chitosan beads by grafting tetra-amine copper(II) (TAC) with chitosan (CS) and utilized the prepared TAC@CS sorbent for phosphate removal. TAC@CS composite beads possessed an enhanced phosphate adsorption capacity of 41.42 ± 0.071 mg/g. The reusability studies of TAC@CS composite beads were carried out using NaOH as the eluent.

Another report [33] examined chitosan beads directly after the saturated adsorption of copper(II) ions (Cu(II)-loaded CS), which was stable and suitable for phosphate adsorption within a wide pH range. The research indicated that the maximum adsorption was achieved at 28.86 mg/g at ca. pH 5.0 (mainly dihydrogen phosphate was adsorbed).

Calcium-modified chitosan microspheres (Ca–CS hydrogel) were studied [34] and showed a high adsorption rate. Adsorption equilibrium of phosphate was achieved within only 20 min, compared with other chitosan hydrogels for phosphate removal. The theoretical maximum phosphate adsorption capacity of Ca–CS gel was 23.7 mg/g by the Langmuir model at neutral pH.

#### 2.1.3. Hydrogels Beads Filled with Carbon or Biochar

Carbon-based materials have been used for years in adsorption processes, e.g., due to their high adsorption capacity (resulting in the presence of various functional groups and developed porous structure) or the possibility of easy surface modification. The most significant carbon-based materials are activated carbon, carbon nanotubes, graphene, and their derivatives. The carbon with chitosan is bound by hydrogen bonds between the hydroxyl and carboxyl groups of the carbon material, and the groups present in the chitosan polymer network [35].

BC is an example of a carbon material used as a filling of chitosan microspheres that are produced under the influence of thermochemical conversion of biomass in organic conditions or free oxygen [36]. The adsorption capacity of such material depends on the production conditions and the origin and composition of the treated biomass [37].

A novel chitosan-modified magnesium-impregnated corn straw biochar and glutaraldehyde cross-linked (CS–MgCBC) was used for the removal of both NH_4_^+^ and PO_4_^3−^ from livestock wastewater [38]. After three desorption processes of CS–MgCBC using only water, the removal rate of PO_4_^3−^ reached 90.3%. From the economic and ecological point of view, the use of sorbent, which requires only clean water for regeneration, is favorable instead of using expensive and sometimes toxic substances. La–SBC–CS microspheres have been reported (previously described in Section 2.1.2 Hydrogels beads modified with metal ions or metal oxides), with a maximum adsorption capacity of ca. 81 mg/g for phosphates [20].

Graphene, or more specifically graphene oxide, is another type of filler. On the surface of graphene oxide, hydroxyl, carboxyl, epoxide, and carbonyl groups are present, producing a hydrophilic character, which is responsible for the formation of electrostatic interactions and hydrogen bonds, e.g., with orthophosphate(V) anions. An example of using graphene oxide as a chitosan filler is described in Section 2.4.

One of the newer carbon-based fillers is carbon nanotubes, which can adsorb pollutants from water matrices. Research conducted by Huang et al. included a sustainable sorbent: chitosan modified with multi-walled carbon nanotubes (CS/MWCNTs) [39]. The maximum adsorption was as high as 36.1 ± 0.3 mg/g and achieved in 30 min at pH 3 and 293 K. The reusability of the CS/MWCNTs composite was investigated in five adsorption–desorption cycles using a desorbing solution of 0.1 M NaOH. The calculated adsorption capacities indicate that the composite maintained good adsorption performance up to the fifth cycle (94% to 98%).

#### 2.1.4. Zeolite and Mineral Composite Hydrogel Beads

The use of minerals as fillers in chitosan sorbents is a perfect combination of two factors that are found in nature and that are non-toxic. Minerals are naturally occurring substances with a solid state of aggregation formed by natural geological processes. The most used mineral-based fillers are clay, aluminosilicates, and zeolites, which have an increased ability to adsorb orthophosphate anions [40].

Jang et al. [41] studied phosphate removal from an aqueous solution using chitosan/Ca-organically modified montmorillonite (CS/Ca–OMMT) beads in batch and fixed-bed column systems. This sorbent was also used for the reclamation of areas exposed to high concentrations of heavy metals. Due to its large surface area and high chemical stability, it behaved as an immobilizer in the chitosan polymer network, which enhanced its mechanical properties and ability to adsorb orthophosphate anions. The maximum adsorption capacity was 76 mg/g after 120 min of contact of the sorbent with the orthophosphate(V) solution, where the initial phosphate concentration was 100 mg/L at 25 °C. CS/Ca–OMMT beads were easily regenerated using 0.1 M NaOH as a desorption agent with more than 83.97% adsorption capacity remaining and desorption removal of 94% after five adsorption/desorption cycles. In another study by Banu et al., the chitosan sorbent was modified with montmorillonite and La^3+^ ions, increasing the adsorption capacity to 129 mg/g after 30 min [42].

Furthermore, modification of the sorbent with zeolites has been used for several dozen years for the adsorption of various substances. For example, zeolite for the adsorption removal of phosphates was possible via modification of chitosan sorbent with La(III) and ZSM–5 zeolite. The tests were carried out by Salehi and Hosseinfard [43], and the maximum adsorption capacity of 152 mg/g was obtained within 30 min and with a sorbent dose of 0.5 g/L at pH 5. The high adsorption capacity resulted from the high specific surface area of the sorbent (S_BET_ = 158.5 m^2^/g) and the presence of many metal cations, including La^3+^, which increased the affinity of the sorbent towards orthophosphate(V) anions.

To improve the properties of bentonite (bent) and chitosan (CS), various multivalent metal ions, such as Zr^4+^, Fe^3+^, and Ca^2+^, were imprinted on chitosan-supported bentonite (CSBent) [44]. Zr^4+^ modified CSBent sorbent displayed the most efficient phosphate adsorption capacity of 40.86 mg/g.

#### 2.1.5. Magnetic Chitosan Hydrogel Beads

Another approach to modifying chitosan is magnetic chitosan composites (MCS), which are obtained through the introduction of a dispersed phase containing magnetic particles, often of nano-size magnetic nanoparticles (MNPs). MNPsCS show high adsorption efficiencies against anionic impurities in aqueous solutions and a high adsorption rate, which is due to the increased interaction of electrostatic forces between the positively charged sorbent surface and negative phosphate ions.

MNPs consist of ferro- and para-magnetic elements, i.e., iron, nickel, cobalt, or their oxides or alloys. These particles interact with the magnetic field and are available in many sizes, including nanoparticles or microparticles. Particles with a size of nanometers (1–100 nm) have gained popularity due to their large specific surface area, the possibility of obtaining a high degree of dispersion, and a high surfaCe–area-to-volume ratio. The combination of these advantages results in an impressive adsorption capacity, making them highly sought after for various applications [45].

Additionally, due to these properties, chitosan sorbents modified with magnetic particles have been used for phosphate adsorption. Inorganic magnetic particles (e.g., Fe_3_O_4_, γ-Fe_3_O_4_, Al_2_O_3_, NiO, ZrO_2_, NiFe_2_O_4_, CoFe_2_O_4_, etc.) are part of the magnetic core or are uniformly dispersed in the chitosan structure. Iron oxides are often used to produce magnetically modified sorbents due to their biocompatibility, strong superparamagnetic properties, low toxicity, and ease of synthesis [46,47].

Kumar and Viswanathan [48] reported Fe_3_O_4_ iron oxide nanoparticles as a chitosan filler. Magnetic chitosan was prepared using both FeCl_3_·6H_2_O and FeCl_2_·4H_2_O salts as precursors of Fe_3_O_4_ nanoparticles, and then the obtained MCS was grafted to form amine-functionalized magnetic chitosan (AFMCS) composite beads. The use of magnetic fillers increased the adsorption capacity of phosphates to 43 mg/g compared to chitosan without modifications. The regeneration performance of AFMCS composite beads, using 0.25 M NaOH as a desorbing solution, was observed up to six cycles, with a desorption efficiency of 98% in the first cycle.

The results on the utilization of MNPsCS beads dried at room temperature are presented in ref. [49]. The batch experiments indicated that phosphate removal by the composites was relatively constant at pH levels of 5.0−9.0, and the adsorption–desorption experiments demonstrated the successful regeneration of sorbent with 0.005 M NaOH solution and the possibility of reusing for phosphate removal. A pilot-scale field experiment showed that the percentage removal of total phosphorous (T-P) in the adsorption column was 52.3% with a phosphate removal capacity of 0.059 mg/g, whereas the effluent T-P concentration was in the range of 0.010–0.028 mg/L.

Fe(III)-doped chitosan (CS–Fe) and cross-linked Fe(III)-chitosan (CS–Fe–CL) composites were developed for the removal of phosphates from an aqueous solution by Zhang et al. [50]. The maximum phosphate adsorption capacity for CTS-Fe and CTS-Fe–CL was 15.7 and 10.2 mg/g at 303 K, respectively. The saturated sorbents of CTS-Fe and CTS-Fe–CL were treated with 0.5 M NaOH solution for 60 min to desorb phosphate with an efficiency of more than 80%.

Karthikeyan et al. [51] used Fe_3_O_4_ nanoparticles as a filler in chitosan modified with kaolin (chitosan-encapsulated magnetic kaolin beads). They found that the introduction of MPs into a modified chitosan composite with a kaolin mineral developed magnetic and, more specifically, paramagnetic properties. Hence, the solid phase could be easily separated from the liquid phase using an external magnet. The capacity of the monolayer for this sorbent was ca. 92 mg/g.

Lanthanum(III) ions were also added to the MCS composite [52]. La–chitosan magnetic spheres showed a high adsorption rate for phosphate at a pH of 3.0. The maximum adsorption capacity of 33.04 mg/g was achieved, which was obviously higher than that of MCS without lanthanum.

In the study by Cui et al., a granular composite sorbent (LC–CS–Fe) from acid-leached carbon waste using chitosan and FeCl_3_ was synthesized and showed a great affinity for phosphate removal. The maximum adsorption capacity of LC–CS–Fe predicted by the Langmuir model for phosphate was 62.72 mg/g. LC–CS–Fe sorbent had good stability and reusability during adsorption–desorption cycles [53].

### 2.2. Chitosan Nanofibers

Nanofibers are another group of chitosan forms that are of interest. They possess an extremely high surface area that contributes to high adsorption capacities, which is important for their future industrial applications. Many publications are focused on the modification of chitosan nanofibers, which are obtained by a popular technique—electrospinning. Similarly to chitosan hydrogels, nanofibers can also be divided according to their modification type. A distinction can be made between modifications with metal ions and metal oxides, nanoparticles, carbon-based organic compounds, and minerals. However, the number of publications on phosphate removal by this method is not extensive compared to hydrogels, which is due to the need for suitable apparatus but also because nanofibers were discovered later than the formation of laboratorY–prepared beads.

An interesting study carried out by F. Bozorgpour et al. [54] employed Al_2_O_3_ and Fe_3_O_4_ nanoparticles for the adsorptive removal of phosphates. Two forms of sorbent were compared namely bead and nanofiber forms. A larger specific surface area (S_BET_ = 251.7 m^2^/g) was obtained for the composite in nanofiber form compared to the composite in bead form (S_BET_ = 72.3 m^2^/g). This resulted in increased capacity of the monolayer from 62 mg/g for beads to 135 mg/g for nanofibers.

In 2021, Palansooriya et al. [36] showed that the modification of chitosan with BC increased the phosphate adsorption capacity to a certain extent, depending on the conditions of biochar production. Thus, BC obtained whilst in contact with CO_2_ increased the adsorption capacity of the composite to 19.2 mg/g compared to that obtained in contact with N_2_ (q_m_ = 16.2 mg/g).

In another report, the application of the coating chitosan technique on flexible nanofibrous membranes modified with ZrO_2_ and SiO_2_ nanoparticles (ZrO_2_/SiO_2_ NM) was examined, which increased the adsorption capacity of orthophosphate(V). The adsorption capacity determined for this composite reached 58 mg/g and was determined, according to the Langmuir model, within ca. 60 min at a phosphate concentration up to 10 mg/L. After the adsorption process, the composite was treated with 0.1 M NaOH to desorb the phosphate ions and potentially achieve their recovery. The ability of the composite to be reused for five adsorption–desorption cycles was also tested. It was confirmed that ZrO_2_/SiO_2_ NM sorbent could be reused without decreasing the adsorption capacity [55].

Richard et al. [56] showed that chitosan could be cross-linked with other polymers, including polyacrylamide, in the presence of glutaraldehyde and used as a bed in SPE columns. The composite had a maximum adsorption capacity of 392 mg/g for 60 min for 0.05 g of the composite in 20 mL of phosphate solution. Regeneration of the deposit was carried out using 2 M HCl.

### 2.3. Chitosan Pellets and Flakes

One of the less frequently mentioned but equally important forms of chitosan used to remove contaminants, including phosphate anions, are pellets or flakes. A comparison of the adsorption properties of chitosan and chitin flakes for phosphate removal showed that chitosan, which is a chitin derivative, had greater adsorption properties [57]. The maximum capacity of the monolayer determined for chitosan was 6.64 mg/g, while for chitin, it was only 2.09 mg/g. The reason for this difference is probably an increase in the deacetylation degree and the presence of active free amino groups on the surface of chitosan, which have a high affinity for orthophosphate anions, especially in the protonated form NH_3_^+^ (at low pH) according to the mechanism described in Section 3. This capacity was determined for a phosphate concentration of 0–25 mg/L after 40 min for chitosan and 20 min for chitin.

Thongsamer et al. [58] investigated the use of chitosan-impregnated coconut shell biochar pellets, where the maximum adsorption capacity was 4.7 mg/g relative to phosphates. The advantage of using chitosan impregnation was to obtain higher phosphate removal rates in surface water samples, for which 66% removal rates were obtained. This allowed the use of this type of sorbent for surface water treatment.

Sowmya [59] reported a chitosan/melamine/glutaraldehyde resin. The maximum adsorption capacity was 112.5 mg/g for an initial phosphate concentration of 1000 mg/L. The adsorption process was better described using the Freundlich model. Furthermore, the paper showed that the sorbent could be used multiple times, both in the batch and column modes. The regeneration study indicated that after 10 adsorption–desorption cycles, the removal efficiency of orthophosphate anions did not decrease, which was a great advantage of using such sorbent in water treatment. Desorption was carried out using 0.025 M NaCl; in the case of the column experiment, complete removal of phosphate ions occurred after washing with 450 mL of NaCl solution at the concentration specified above.

Ref. [60] described a novel pilot-scale technology using a FILTRAFLOTM-P reactor to recover phosphate (PO_4_^3−^) from wastewater effluent through a filtration/adsorption process in a rural setting. The sorbent was used in granule form, creating a novel chitosan-calcite material (CCM). The parent material of CCM was brown crab carapace, composed mainly of calcite and chitin, and is a common waste bY–product from the seafood industry worldwide. High phosphate removal was achieved at low phosphate concentrations, resulting in the residual effluent phosphate level being below 1 mg/L (EU limit for sensitive water bodies) and at higher PO_4_^3−^ levels, more than 50%, respectively. Furthermore, P-enriched CCM could be used as a potential soil amendment.

### 2.4. Ground and Fine Chitosan Forms

Numerous publications have reported the adsorption of phosphates on chitosan sorbents in dry and ground forms in recent years. Despite some inconveniences related to the separation of the solid from the liquid phase after the phosphate adsorption process, the obtained results have encouraged further research.

Lanthanum(III) and other metal ions have been used not only to obtain chitosan hydrogel granules but also to obtain chitosan composites in their fine form. Non-cross-linked lanthanum-chitosan (La–CTS–0X) and glutaraldehyde cross-linked lanthanum-chitosan (La–CTS–1X/2X) composites were prepared and studied for phosphate removal from wastewater by batch adsorption experiments [12]. The maximum phosphate adsorption capacity achieved was 57.84 mg/g for La–CTS–1X, whereas the initial P concentration was 50 mg/L, which was considerably larger than the adsorption capacities of 47.28 and 31.01 mg/g for La–CTS–0X and La–CTS–2X, at pH 6, respectively. The study found that lanthanum chitosan adsorbed phosphate, forming LaPO_4_, which was difficult to dissolve in an alkali solution. To remove the phosphate, hydrofluoric acid was used as a desorbing agent, and the recovered fluorine was treated with 0.1 M sodium hydroxide solution. Even after three cycles of adsorption and desorption, La–CTS–1X retained an 80% phosphate removal rate.

Modification of wheat straw biochar with chitosan, quaternary ammonium salt, and lanthanum for the purpose of enhanced phosphate removal was reported by Huang et al. [61]. A maximum adsorption capacity of 109 ± 4 mg/g was obtained. The phosphate removal performance of the composite was not significantly affected over a broad range of temperatures (15–45 °C) and pH (2.5–7) after 30 min and reached an adsorption equilibrium. Desorption was performed over three cycles with 0.1 M NaOH solution.

Phosphate adsorption using low-cost sorbents consisting of lanthanum compounds, bentonite, and chitosan has been reported [40,62]. Lanthanum-doped bentonite–chitosan composite (La–BCS) was prepared using a simple, easy, and scalable method that combines direct calcination and subsequent blending. The optimal sorbent content of 5%(wt.) chitosan (La–BCS–5) rapidly reached equilibrium after 20 min and provided 93.2% adsorption efficiency (initial phosphate concentration, 50 mg/L) and as high as 99.7% was removed in 5 min under low phosphate concentration (2 mg/L) [40]. Notably, when calcination and blending were not conducted, the outcome was much worse, where the adsorption equilibrium was reached within 30 min, and the adsorption capacity was 15.5 mg/g, with an initial phosphate concentration of 50 mg/L. Despite achieving a medium adsorption capacity, La–BCS had a high selectivity towards orthophosphate anions in a mixture containing other ions such as SO_4_^2−^, HCO_3_^−^, NO_3_^−^, or Cl^−^, which are commonly found in large quantities in urban water bodies. The desorption process was carried out with 1 M NaOH on a shaker for 24 h. Five adsorption/desorption cycles were carried out, resulting in the adsorption removal efficiency remaining at ca. 80% [62].

In other studies, Kumar and Viswanathan [63] investigated the adsorption of phosphates using Zr^4+^ ions in the form of zirconium oxyhydroxide with kaolin (a sedimentary rock with the main composition of kaolinite, quartz, and mica). In these studies, due to the high specific surface area (S_BET_ = 85.5 m^2^/g), the highest adsorption capacity (41 mg/g) was obtained by the sorbent produced via the hydrothermal method, and the adsorption equilibrium was reached within 30 min of the process.

In certain cases, chitosan was employed to pre-modify other materials, e.g., diatomite, where ZIF–8 was anchored to the surface of the diatomite solidly and uniformly. The pre-modified diatomite composited with ZIF–8 (CZD) removed 85% of phosphate rapidly and efficiently within the first 10 min. The modified diatomite adsorbed up to 13.95 mg/g of phosphate at 25, 35, and 45 °C. CZD effectively removed over 98% of phosphate, reaching 100% at 45 °C and an optimal pH of 5.81 [64].

Several papers employed MNPs for the synthesis of finely granulated phosphate sorbents. A novel triethylene tetramine-functionalized magnetic graphene oxide chitosan composite material (TETA–MGO/CS) with a high adsorption capacity for phosphate was prepared by Wang et al. [65]. The maximum adsorption capacity of TETA–MGO/CS at 298 K calculated from Langmuir isotherm was 353.36 mg/g compared to 314.83 mg/g obtained for MGO/CS sorbent without triethylene tetramine (TETA) functionalization. The observed increase was attributed to the rise of amino groups following the functionalization of MGO by TETA. Moreover, after magnetic separation, phosphate ions adsorbed onto TETA–MGO/CS could be desorbed with 1 M of sodium hydroxide and sodium chloride mixture solution. The adsorption and desorption efficiency of phosphate on TETA–MGO/CS were above 80% for the first three cycles and above 60% for the next three cycles.

Fu et al. [66] used magnetic nanoparticles to create a ground chitosan sorbent for the removal of phosphate. They synthesized Fe_3_O_4_/CS/PEI magnetic nanoparticles with a polyethyleneimine-grafted chitosan core-shell. The highest phosphate adsorption rate of 50.8 mg/g was achieved at a pH of 4.0 and temperature of 25 °C. MNPs were recovered using an applied magnetic field, and over 90% of the phosphate was desorbed with 0.05 M of NaOH solution. The five-cycle reusability tests showed that Fe_3_O_4_/CS/PEI particles had promising potential for future applications.

Han et al. [67] prepared a magnetic Fe_3_O_4_ doped lignin-chitosan-based microspheres (CS–Li@ Fe_3_O_4_) that showed a spherical structure with a diameter of 5–40 μm. CS–Li@ Fe_3_O_4_ reached a maximum adsorption capacity of 98 mg/g at a pH range of 2–10. It is crucial to note that CS–Li@ Fe_3_O_4_ desorbed by alkaline solution (pH 10) retained excellent adsorption capacity after six adsorption/desorption cycles and demonstrated remarkable recyclability and stability.

Another method of modifying chitosan to obtain a fine and dry sorbent is chemical derivatization using caproyl and palmitoyl fatty acid chains. The phosphate adsorption studies showed enhanced adsorption efficiencies for caproyl chitosan (63%) and for palmitoyl chitosan (71%) in aqueous solutions containing phosphate at pH 6.2. However, both raw and modified chitosan sorbents showed increased removal efficiency at an optimal solution pH of 4. The adsorption kinetics studies performed on palmitoyl-chitosan indicated that a contact time of ca. 30 min was required to reach equilibrium at solution concentrations ranging from 10–20 mg/L. Co-existing anions such as nitrates and sulfates negatively affected the phosphate removal efficiency of modified sorbents in aqueous solutions due to the affinity of these anions to the active sites on the modified chitosan [68].

### 2.5. Chitosan Nanoparticles and Chitosan Sponge

Chitosan nanoparticles (CSNP) have a higher adsorption capacity than regular chitosan. Chitosan can be easily modified on the nanoscale by cross-linking with sodium tripolyphosphate. To prevent agglomeration of the nanoparticles, it is advised to impregnate them on a suitable polymer surface. One such polymer is polyurethane foam (PUF), which possesses desirable qualities, including flexibility, porosity, and the capability to bind with nanoparticles through its carbamate group (-N(H)COO-) [69].

Sasidharan et al. [70,71] tested different types of polyurethane foam impregnated with chitosan nanoparticles (PFC). The experimental studies showed that at optimized operating conditions, PFC removed 26.15% of phosphate within 6 h from the synthetic greywater containing a phosphate concentration of 155 mg/L [71]. After additional impregnation of PFC with silver/silver oxide nanoparticles (AgNP), a new PFCA sorbent was obtained, which removed up to 61.24% of phosphates from the influencing phosphate concentration of 50 mg P/L in its propitious conditions. Even after being reused seven times, PFCA removed 20.58% of phosphate [70].

In a study by Salehi and Hosseinfard [72], CSNP sorbent was further modified with graphene oxide (GO) and zirconium ions to obtain Zr–nanochitosan/graphene oxide composite (NC@GO/Zr). The tests confirmed the high adsorption capacity of graphene oxide and chitosan nanoparticles, which resulted in an adsorption capacity of NC@GO/Zr towards phosphates equal to 172.4 mg/g. The addition of the zirconium ions was intended to improve the adsorption selectivity towards orthophosphate(V) anions. In the same year, Salehi and Hosseinfard [43] modified chitosan on the nano-size scale with lanthanum(III) ions and ZSM–5 zeolite. They found that the sorbent produced from zeolite, CSNP, and lanthanum(III) had a high adsorption capacity of 152 mg/g due to its large specific surface area and the presence of metal cations, including La^3+^. These cations increased the sorbent’s affinity for orthophosphate(V) anions.

Yang et al. study [73] developed a sponge filter (SF) from lanthanum-flocculated graphene oxide (LaFGO) and chitosan to separate phosphate from aqueous solution through a chemical filtration process. The filtration capacity on phosphate was 13.12 mg/g, where the filtrated phosphate was adsorbed in the material and transformed to LaPO_4_ precipitate.

### 2.6. Chitosan as a Direct Bio-Flocculant

Chitosan and chitosan-based products have been applied as bio-flocculants for the removal of specific and dissolved pollutants by direct bio-flocculation process. Coagulation and flocculation processes are used during industrial and municipal water treatment to remove suspended particles. Metal salts are common coagulants, whereas flocculants are synthetic organic polymers. Direct flocculation is conducted using water-soluble, ionic organic polymers without classical metal-based coagulants, thus limiting water pollution. For example, 97% phosphate removal was observed over a wide pH range using (3-chloro-2-hydroxypropyl)trimethylammonium chloride grafted onto carboxymethyl chitosan [74].

In the study described in ref. [75], the bioreactor containing the culture of *cyanobacterium* and chitosan as the flocculant was investigated for the removal of phosphates. The efficiency of chitosan flocculation was over 90% even with high cell concentration in the photobioreactor, and the resulting biomass contained high phosphate content. The optimal flocculation process was accomplished by adjusting the pH to 7.2 before the addition of 20 mg/L chitosan, followed by a pH adjustment to 7.5. The process of phosphate removal with wastewater was repeated 12 times with an average phosphate removal efficiency of 98.6 ± 0.8%.

Zhao et al. [76] discovered that chitosan had another benefit in that it affected the diversity and composition of phosphate-solubilizing bacteria in wastewater treatment. Chitosan acted as a carbon source, leading to an increase in the population of certain phosphate-solubilizing bacteria, such as *Pseudomonadaceae*, *Rhodocyclaceae*, *Bacillaceae*, and *Enterobacteriaceae*, in the activated sludge. Additionally, chitosan enhanced the enzymatic activity, such as dehydrogenase and phytase, which promotes the dissolution of organophosphorus. Hence, chitosan contributed to the improvement in dephosphorization in wastewater treatment.

### 2.7. Summary of Chitosan Sorbents for Phosphate Removal

An issue that arises with the utilization of chitosan sorbents for phosphate removal is the rather moderate adsorption capacity. Much higher adsorption capacities are observed for chitosan sorbents modified with metal ions (up to 200 mg/g), carbon materials, e.g., nanotubes or graphite, nanoparticles, nanofibers, or their combinations (even over 300 mg/g). Unfortunately, such systems incur higher costs associated with the purchase and production of expensive materials for fillings, e.g., lanthanide salts or graphene oxide. The structure of sorbents also affects the adsorption efficiency. It seems that nanofibers or nanoparticles of chitosan composites have great potential in adsorption processes due to a larger specific surface area; however, the cost of their production is currently the limiting factor for large-scale applications.

Thus, the above-mentioned types of modified sorbents have certain advantages and disadvantages. In certain cases, despite low adsorption capacity, they contain cheap and ecological fillings, while others are quite the opposite. Therefore, the selection and use of a specific sorbent are not clear, and research is still focused on exploring those with the highest adsorption capacity. Table 1 summarizes the adsorption capacities and the most important parameters of the adsorption process of the chitosan sorbents described above, divided into their various forms and modifications.

## 3. Mechanism of Phosphate(V) Adsorption on Chitosan Sorbents

Gaining a comprehensive understanding of the mechanism of adsorption is problematic and requires advanced analytical equipment and experienced researchers. In the works presented above, attempts were made to explain how the adsorption of phosphate species occurs on the surface of the tested chitosan sorbents.

### 3.1. Non-Modified Chitosan

The adsorption mechanism of phosphate ions on CSHs is based on electrostatic and ion-exchange processes that contribute to the adsorption of HPO_4_^2−^ by chitosan materials. In addition, the adsorption of HPO_4_^2−^ possibly involves hydrogen bonding between the polar functional groups of chitosan (-OH, -NHR; where R is acetyl or H) with the donor-acceptor groups of HPO_4_^2−^ to variable extents that depend on the hydrophile-lipophile balance of the bead surface, according to the nature of the cross-linking and composition of the chitosan beads [16].

The pH level of the solution plays a crucial role in the ability of CSHs to adsorb orthophosphates due to the attraction between the negatively charged phosphate and positively charged amino groups on CSHs. It is known that orthophosphates can be identified as different ionic forms in the solution, depending on its pH. For pH values below 6, H_2_PO_4_^−^ ions predominate; at pH above 6, HPO_4_^2−^ ions are mainly present, and above pH 9, PO_4_^3−^ ions dominate (Table 2).

Regarding the surface of hydrogels, as the pH of the solution decreases, the number of positively charged -NH_2_ groups increases, which enhances the capacity to adsorb anions such as H_2_PO_4_^−^. Thus, CSH is the most effective at adsorbing phosphates in acidic solutions, particularly at pH 4. Above a pH of 5, CSH’s ability to absorb phosphates decreases. This is due to the chitosan’s surface becoming negatively charged under alkaline conditions, allowing the repulsion of the anionic phosphates and preventing their adsorption. Additionally, the low effectiveness of PO_4_^3−^ adsorption at high pH values may be due to the competition between i–PO_4_ and OH^−^ ions for the adsorption centers of CS [13]. Studies on the effect of pH on the adsorption of orthophosphate(V) anions have resulted in an interpretation of the adsorption process related to the electrostatic attraction, indicating that physical adsorption occurs [57].

**Figure 7:**
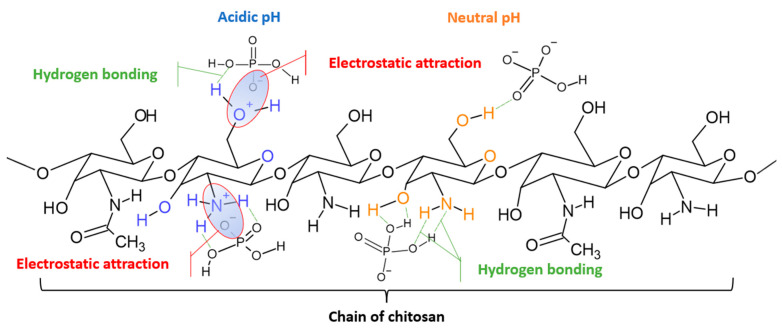
Probable mechanisms of orthophosphates(V) bonding onto chitosan (electrostatic attraction in red color, hydrogen bonding in green color). Source: own elaboration.

The adsorption of phosphates onto CS Is also supported by hydrogen bonds following the electrostatic interactions between the cationic CS sorbent and the anionic adsorbate (Figure 7). Herein, we distinguish two possible combinations. The first occurs when the oxygen atom of the phosphate and the hydrogen atom of the amino group of CS are linked through a hydrogen bond. The second is when this hydrogen bond occurs between the hydrogen of the phosphate anion (H_2_PO_4_^−^ or HPO_4_^2−^) and the nitrogen of the CS amino group. Both mechanisms are possible during phosphate adsorption onto CS between pH 2.0 and 12.5, which was observed and shown in Figure 7.

As mentioned earlier, according to reports, the non-cross-linked CSHs showed the highest adsorption efficiency at pH 4. At lower pH values (2–3), it swelled and dissolved, resulting in a diminished ability to bind phosphate. In turn, the chitosan hydrogel cross-linked with epichlorohydrin was the most effective in binding phosphate anions at pH 3, in which solution it was not dissolved [14].

### 3.2. Metal-Based Chitosan

The metal-chitosan composites have significantly enhanced the adsorption capability towards phosphate ions, which can be explained by the Pearson HSAB principle. The strong attraction between the metal ions and phosphate species, as well as the electrostatic interactions between metal ions and the protonated groups of CS (-NH_3_^+^ and -OH_2_^+^) with phosphate ions in acidic pHs, have been observed. Furthermore, the hydrated form of metal oxide, such as α–FeO(OH), may interact with phosphate ions by inner-sphere complexation. When the biopolymeric granules possess alginate in their matrix, which contains carboxyl groups, these groups are protonated in strongly acidic conditions and, thus, can interact with the phosphate ions by electrostatic attraction. Together with the possible ligand exchange between the (-OH) group of M-OH and phosphate ions under alkaline conditions are the key factors contributing to the increased adsorption capability [17,35]. In the case of the hydrogel immobilized with La(OH)_3_ nanoparticles, phosphate adsorption had two kinds of mechanisms: the electrostatic interaction from -N^+^(CH_3_)_3_ groups and the ligand exchange from immobilized La(OH)_3_ nanoparticles or from some combined anions, e.g., Cl^−^ in sorbent by i–PO_4_ groups. The strong chemical interaction between La atoms and phosphate ions through La–O–P bonds was confirmed [18,19]. Probably, lanthanum-modified chitosan adsorbed phosphate and formed LaPO_4_ [12].

The mechanistic study of phosphate adsorption with the use of chitosan hydrogel beads modified with Zr^4+^ ions confirmed that electrostatic attractions and ion exchange reactions between phosphate ions and modified CS played an important role in phosphate adsorption. However, it has been proven that metal oxides, such as ZrO_2_, exhibit strong phosphate ligand adsorption through the formation of intra-spherical complexes [22]. Various reports have explained these phenomena as ligand exchange between phosphate and hydroxyl via direct coordination of phosphate, a Lewis base that donated electrons to zirconium atoms that have vacant orbitals and may accept electrons [24]. Therefore, it should be assumed that multiple mechanisms, such as electrostatic interaction, ligand exchange, and ion exchange, participate in the phosphate removal by zirconium CSH sorbents [23]. Similar conclusions were drawn in studies on chitosan hydrogel modified with Ce^3+^ ions [26,27].

The characteristic of the chitosan hydrogel beads modified with the less valuable metals, i.e., Zn^2+^, Cu^2+^, or Ca^2+^, indicate that the metal(II) loaded at the various functional sites adsorb phosphate by electrostatic attraction, and the formed metal ion-CS ((CTS-NH_2_-M)^n+^) complex can be explained according to Lewis acid-base theory. Here, the metal ion (M^2+^) plays the role of the acid by accepting the electron pairs provided by CS as the base. Furthermore, Cl^−^ ions present in the sorbents (as evidenced by EDAX) can be exchanged for phosphate anions [29,30]. However, a different mechanism occurs in the case of phosphate adsorption on chitosan modified with Ca^2+^ ions. Reports showed that the calcium-ions-modified chitosan was significantly different from zinc or lanthanum-modified chitosan in the loading principle. During the coagulation of Ca–CTS gel, the coordination ability of Ca^2+^ and chitosan was relatively small, and, therefore, Ca(OH)_2_ was attached to the surface of chitosan by in situ precipitation. In contrast to the mechanism of Zr^4+^, Ce^3+^, and La^3+^, Ca–CTS hydrogel removed phosphates by surface precipitation in the form of CaHPO_4_⋅2H_2_O and Ca_5_(PO_4_)_3_OH (determined by XRD analysis). Hence, Ca(OH)_2_ played a primary role in the process of phosphate adsorption on Ca–CTS sorbent, in which the mechanism was related to the precipitation conversion between Ca(OH)_2_ and CaHPO_4_⋅2H_2_O and Ca_5_(PO_4_)_3_OH [34]. Furthermore, when calcite was introduced into the chitosan matrix, the surface microprecipitation of CaHPO_4_, Ca_3_(PO_4_)_2_ followed interactions between P and -Ca–CO_3_ groups that were postulated as the main i–PO_4_ adsorption mechanisms (evidenced according to XRD, XPS, and EDX elemental mapping) [60]. Similar conclusions were formulated in studies using Mg^2+^ ions, where i–PO_4_ adsorption on chitosan-modified magnesium-impregnated corn straw biochar and glutaraldehyde cross-linked (CS–MgCBC), resulting in the formation of struvite. It remained in the BC, which could be used as phosphate fertilizer without causing secondary pollution to the environment [38].

### 3.3. Carbon-Based Chitosan

In the case of the removal of phosphates on chitosan modified with various types of carbon, e.g., with GO or nanotubes, the process mechanism consists of the physical adsorption via electrostatic attraction, which is independent of chitosan’s form: beads, nanofibers, powder, pellets, etc. The electrostatic attraction between the positively charged surfaces of sorbent and phosphate anions was the main mechanism for phosphate adsorption when the filler of chitosan was multi-walled carbon nanotubes in the form of beads [39], nanofibers with biochar [36], coconut shell biochar pellets [58], and granulated magnetic graphene oxide [65].

To increase the efficiency of the process, the sorbents were additionally modified with metal ions. Then, the adsorption of the phosphates proceeds according to the mechanism given for the Metal-based chitosan section discussed above. It was reported that for chitosan modified with biochar and polyvalent metal ions, such as Zr^4+^, La^3+^, Fe^3+/2+^, and Zn^2+^, phosphate adsorption proceeded according to a complex mechanism consisting of electrostatic interaction, ion or ligand exchange and complexation [20,61]. In the case of a sponge filter modified with lanthanum-flocculated graphene oxide (LaFGO), phosphate separation was a chemical process in which phosphate ions from the solution were trapped and transformed to LaPO_4_ [73]. However, when the modifying ions were Ca^2+^ or Mg^2+^, the adsorption process was caused by electrostatic attraction and precipitation combined with the conversion of precipitates on the chitosan surface [34,38].

### 3.4. Mineral-Based Chitosan

The use of mineral filling of chitosan matrix with, e.g., montmorillonite, bentonite, or zeolite enhanced its ability to adsorb i–PO_4_ owing to the large surface area and the presence of new chemical components, e.g., calcium compounds, aluminates or aluminosilicates. Among the overmentioned adsorbents, calcium ions on the surface of the beads play a dominant role in capturing phosphate ions through surface precipitation or complexation [41]. In addition, the introduction of iron(III) or zirconium(IV) ions caused complex adsorption, supported by complexation and ion exchange reactions [43,44], while lanthanum(III) ion filler favored H-bonding [42] or chemical bonds generated by the formation of inner layer complexes [62]. The electrostatic attraction was the primary mechanism of phosphate removal using pre-modified diatomite CZD [64]. It should also be mentioned that a favorable pH range of 3–7 was observed for phosphate adsorption. In alkaline media, the adsorption capacity diminished due to the electrostatic repulsion between the phosphate ions and the negative composite surface.

### 3.5. Magnetic-Based Chitosan

The use of magnetic filling increased the adsorption capacity of i–PO_4_ and facilitated the separation of the sorbent from the liquid employing an external magnet; hence, such a solution in the preparation of chitosan sorbents appeared in many publications on this discussed topic. Generally, when Fe_3_O_4_ nanoparticles were used for phosphate removal, the adsorption mainly followed electrostatic attraction and surface complexation mechanisms [48]. In acidic conditions, phosphate and chitosan functional groups (-NH_2_ and -NH_3_^+^) reacted through electrostatic attraction, while iron hydrolysates bonded through ligand exchange. The hydrolysis reaction of Fe(III) released a significant amount of hydrogen ions, which enhanced the protonation and electrostatic attraction under acidic conditions. Importantly, adsorption could also be carried out under more alkaline conditions (up to pH 9), where the electrostatic attraction was weakened due to an increase in the amount of OH- ions, and ligand exchange became the dominant mechanism for phosphate adsorption [50].

When using chitosan-encapsulated magnetic kaolin beads (MK-CS) to remove phosphate ions, the metal, metal oxide, and amino groups in the beads attract the negatively charged ions through electrostatic force. Hydrated metal oxides such as Si(OH)^3+^, Mg(OH)^+^, Fe(OH)^2+^, and Al(OH)^2+^ form a network with i–PO_4_ through surface complexation [51]. The same mechanism occurs for Al_2_O_3_ together with Fe_3_O_4_ MNPs. Additionally, -OH groups in the MK-CS exchange with phosphate ions in the aqueous solution [54]. The addition of lanthanum ions to chitosan magnetic spheres promotes a strong attraction and increases the capacity for the adsorption of i–PO_4_ via electrostatic attraction and ligand exchange. Both -NH_2_ and -OH groups participate in the coordination process [52]. In contrast, the addition of waste carbon triggers the hydrogen bonding mechanism [53].

An adsorption mechanism based solely on Coulomb attraction was reported for Fe_3_O_4_/CS/PEI magnetic nanoparticles with a polyethyleneimine-grafted chitosan core-shell and a magnetic Fe_3_O_4_ doped lignin-chitosan-based microspheres (CS–Li@Fe_3_O_4_). The free amino and hydroxyl groups on the sorbent surface were protonated to -NH_3_^+^ (major) and -OH_2_^+^ (minor) groups under low pH conditions. In this regard, the surface of sorbents had a strong affinity to H_2_PO_4_^−^ anions through electrostatic action [65,66].

### 3.6. Polymer-Based Chitosan

In the case of chitosan/melamine/glutaraldehyde sorbent, the adsorption process is mainly related to the occurrence of ion exchange phenomena. The ammonium groups present in the sorbent have Cl^−^ ions, which are exchanged for orthophosphate(V) anions, so high phosphate adsorption capacities are observed. Reports have shown that the effect of pH on the adsorption capacity obtained within the range of 3–10 remained constant, confirming that the adsorption mechanism was based on ion exchange and not on electrostatic interactions [59]. In contrast, chitosan nanofibers cross-linked with other polymers, including polyacrylamide in the presence of glutaraldehyde, adsorbed phosphates according to a complex mechanism consisting of electrostatic interactions, hydrogen bonds or weaker van der Waals interactions [56].

**Figure 8:**
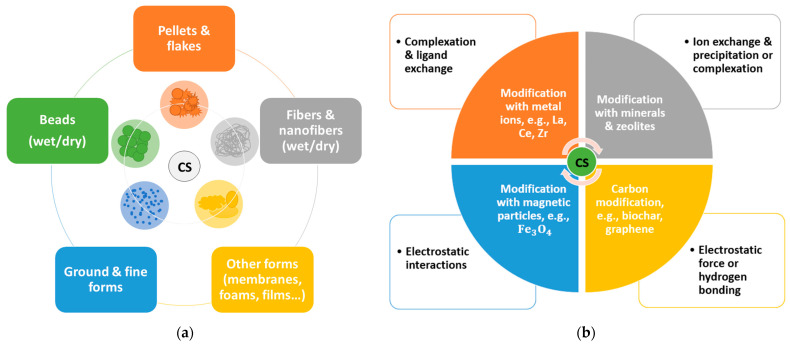
Division of chitosan into (**a**) different structural forms and (**b**) different modifications influencing the adsorption mechanism. Source: own elaboration.

Therefore, in terms of the phosphate ions adsorption mechanism on chitosan sorbents, it can be stated that the method of binding these ions on the surface of the sorbents, at least in the majority of the tested sorbents, has yet to be clearly determined. Nevertheless, based on the collected data, it is known that the affinity of orthophosphate anions to the chitosan sorbent is associated with the occurrence of several types of interactions between the sorbate and the sorbent, involving functional groups in chitosan. These interactions include ion and ligand exchange, complexation, and precipitation, as well as hydrogen or van der Waals bonds. However, the physical interaction as a result of electrostatic attraction between the positively charged surface of the sorbents and the negative phosphate ions in the acidic medium is usually the first stage of the mechanism. However, the predominance of some interactions over others depends on the incorporated modification of the sorbent, for example, on the type of filling (Figure 8).

## 4. Desorption of Phosphates(V) from Chitosan Sorbents

As mentioned above, chitosan sorbents may have great potential in the protection of the natural environment, i.e., in wastewater treatment plants, e.g., for the final treatment of wastewater after chemical methods, and in surface water purification at risk of eutrophication. However, it is important to remember an important aspect of a circular economy, which is the recovery of phosphorus. Phosphorus is an element classified as a critical raw material; therefore, its recovery should be introduced into all possible processes, including the revitalization of water reservoirs or wastewater treatment in wastewater treatment plants. Revitalization of surface waters that often contain high concentrations of nutrients, including phosphorus, mainly as a result of agricultural runoff from intensively fertilized soils, is an important source of phosphorus that has yet to be properly exploited.

For phosphorus recovery, it is necessary to examine the process of its desorption from modified chitosan sorbents. Unfortunately, studies on the process of desorption and recovery of phosphorus from such saturated sorbents are rare. From the literature review mentioned earlier (in Section 2), research on desorption is not well developed, and repetitive concepts are commonly reported, some of which are not always successful. In this section, we explore phosphate desorption research from saturated chitosan sorbents.

The most commonly published substance for phosphate desorption is sodium hydroxide. It is abundantly available and inexpensive. The desorption of orthophosphate(V) from unmodified chitosan hydrogel and epichlorohydrin-cross-linked chitosan hydrogel using sodium hydroxide was effectively carried out with removal rates of over 95%, and the possibility of phosphorus recovery for 20 cycles. The kinetics of the desorption process was also considered, showing that after ca. 60 min for CSHs and ca. 90 min for CSHs-ECH, a desorption equilibrium state was established [14].

In a paper on the adsorptive removal of phosphates using a glutaraldehyde-modified chitosan hydrogel, a high phosphate desorption capacity from the tested sorbent was also demonstrated. The results showed that phosphate desorption was ca. 100% after nine cycles using a 0.1 M sodium hydroxide solution with a contact time of 60 min. Similar results were also observed using a sodium chloride solution of the same concentration as a desorbing agent [77]. Equally high efficiencies were obtained by Wan et al. in a study of phosphate desorption from a chitosan hydrogel modified with zirconium ions and PVA. They investigated five desorption cycles using 0.05 M sodium hydroxide and achieved 100% desorption. The difference with the use of a lower concentration of NaOH was probably compensated by the pre-use of hydrochloric acid at a concentration of 0.05 M, hence allowing additional ion exchange of orthophosphate(V) anions to chloride anions [78].

In some cases, sodium hydroxide solutions are not sufficient as a desorption agent to remove phosphate from the sorbent with high efficiency, both in the first and subsequent desorption cycles. For example, for the desorption of phosphate from a kaolin-lanthanum-modified chitosan hydrogel, only 70% phosphate desorption was achieved after five cycles using 0.1 M sodium hydroxide [79]. In a study by Ribeiro et al., the percentage of phosphate desorption from an aluminum-magnesium mineral-modified hydrogel using 0.01 M sodium hydroxide was ca. 66% [80].

Salts are less commonly used desorbing agents. Zavareh et al. employed a chitosan hydrogel modified with Cu^2+^ copper ions and iron(II/III) oxide nanoparticles. The role of the desorbing agent was played by copper(II) sulfate(VI) at a concentration of ca. 0.06 M and a contact time of 2 h. The resulting percentage desorption of phosphates carried out for five cycles was ca. 95% [81].

In addition to desorption studies on chitosan hydrogels, the desorption process was also examined for other forms of chitosan sorbents. Wang et al. investigated the use of chitosan coating on flexible nanofibrous membranes modified with ZrO_2_ and SiO_2_ nanoparticles (ZrO_2_/SiO_2_ NM), where at a concentration of 0.1 M NaOH, phosphate desorption of 95% tested for five adsorption/desorption cycles was possible. In this case, the morphology of the sorbent was unchanged, which was confirmed by SEM analysis [55].

The potential of phosphorus recovery using chitosan/melamine/glutaraldehyde resin pellets was tested by desorbing phosphate with a 0.025 M NaCl solution. The reported results showed that the phosphate removal efficiency remained constant after 10 adsorption/desorption cycles. Surprisingly, to obtain a high desorption rate, a rather low concentration of desorbing agent was employed. This was due to the use of a column during the adsorption test and the fact that the phosphate itself was adsorbed on the resin through ion exchange. Therefore, the use of this sorbent in water treatment may possess great potential [59].

The desorption process of another sorbent, Fe_3_O_4_/CS/PEI magnetic nanoparticles with a polyethyleneimine-grafted chitosan core-shell, was investigated using 0.05 M NaOH. This resulted in a high desorption percentage of over 90% for five adsorption/desorption cycles [66]. In one study, chitosan sorbent was tested in a polyurethane foam impregnated with nano chitosan and modified with silver oxide nanoparticles. PFCA showed a phosphate removal capacity of 61%, which was insufficient for possible applications and because of the expensive silver compounds used. A study on adsorption/desorption cycles showed that after seven cycles, the phosphate removal efficiency dropped to ca. 21% [70].

A literature review on the possibilities of phosphate desorption from modified chitosan sorbents shows the need for further investigation of desorbing agents, as their action depends on the type of sorbent and how phosphates interact with the modified sorbent. A significant amount of publications, among others discussed in the previous section, employ sodium hydroxide as a desorbing agent. However, the desired outcome of high phosphorus recovery rates is not always possible. The section detailing phosphate desorption testing did not cover the choice of parameters, i.e., the appropriate desorbing agents, their concentration, or the desorption process time. Table 3 summarizes the results of studies on the process of phosphate desorption from chitosan sorbents.

According to the above literature review and Table 3, it appears that the desorption of phosphate from chitosan hydrogels is strongly influenced by the pH of the desorbing solution, as well as the type of ion used. The effect of this synergy can be explained by the fact that when an alkaline solution is in contact with the sorbent, the surface of the chitosan sorbent assumes a negative charge. This is due to the interaction of the hydroxyl groups with the functional groups of the polymer, according to the reactions below:

-NH_3_^+^ + OH^−^ → -NH_2_ + H_2_O (1)

-OH + OH^−^ → -O^−^ + H_2_O (2)

This results in orthophosphate anions (in alkaline media, HPO_4_^2−^ and PO_4_^3−^ anions predominate) being repelled from the negatively charged sorbent surface, moving away and diffusing deep into the desorbing solution. The type of ionic substance used has a large impact on the effect of desorbing phosphate from sorbents; e.g., NaCl enables the exchange of Cl^−^ ions for orthophosphate anions and detaching the orthophosphate anions. The two interactions presented, electrostatic interactions and ion exchange, are most often described in publications as the main interactions that affect desorption but also the adsorption of orthophosphate(V) anions. As mentioned earlier, the interactions between the sorbent and sorbate can be various, i.e., electrostatic interactions, ion exchange, complexation reactions, van der Waals forces, and hydrogen bonds, which depend on the modification of chitosan [35]. Since these interactions have different energies and thus different durability, it can be assumed that, depending on the modification of the sorbent, one of the aforementioned interactions dominates, which then affects the rate and efficiency of desorption.

## 5. Conclusions

Chitosan sorbent is an ideal choice for water and wastewater treatment because it has many desirable qualities. It is biodegradable, originates from natural sources, is highly abundant, reactive, non-toxic, and has many biological benefits, such as anti-tumoral, anti-microbial, anti-fungal, antioxidant, and anti-inflammatory properties. It is also suitable for removing phosphates, as evidenced by the large number of publications that appear on this topic every year. Already in the unmodified form, e.g., hydrogel beads, it has an affinity for H_2_PO_4_^−^ in an acidic environment, which is due to the presence of protonated amino and hydroxyl groups in the biopolymer network and the positively charged surface. In general, researchers focus on two issues: improving the adsorption properties of chitosan towards phosphate ions through the introduction of substances with physical or chemical affinity to i–PO_4_ into the matrix and improving its physicomechanical properties. The abundance of functional groups of chitosan enables modification by cross-linking and the generation of materials with variable morphology, such as powders or spheres with variable acid stability, mechanical strength, pore size distribution, and hydrophilic-lipophilic balance. These results have allowed for the design of a phosphate adsorption system on an industrial scale using properly selected chitosan sorbent, whose laboratory tests indicate the possibility of achieving high adsorption capacity (up to several hundred mg per gram of chitosan) and high efficiency of phosphate removal (nearly 100%).

However, the analysis displayed in this literature review revealed some doubts or gaps in the area. Firstly, studies of phosphate desorption from saturated chitosan sorbents are not always shown in publications, which may indicate some problems in conducting such examinations. Secondly, desorption studies, which resulted in an effective factor of phosphorus recovery from the sorbent, rarely have a proposed method of handling the obtained phosphate concentrate. Unfortunately, the recovery of phosphorus and the indication of the possibility of its reuse are shown in only three cited articles. Phosphorus—a critical element—is an economically valuable element, while water and sewage can be a secondary source of its acquisition. The third issue is important in terms of the circular economy but also of broadly understood economics. None of the available published papers contain information on the costs incurred when using chitosan as a sorbent in water and wastewater treatment. It would be worth comparing these costs with, for example, commercial strong base anion exchangers or chelating resins in the copper form (phosphate selective) or with Ferrolox, a commercial filtering material for phosphate removal, and then, perhaps, biopolymers could prove to be competitive materials in adsorption processes, also in economic terms.

## Figures and Tables

**Table 1:** Comparison of the adsorption capacity (calculated per dry mass of sorbent) of chitosan sorbents used for orthophosphate(V) removal.

Form of Chitosan	Modification Type	Name of Sorbent	q [mg/g]	t_eq(s)_ [min]	pH, T [K]	C_P-PO4_ [mg/L], Sorbent Dose [g/L]	Ref.
Hydrogel beads	without modification	CSH	15.72	60	4, 295	3–300, 1	[13]
		CSHs	18.85	60	3, 295	1–100, 1	[14]
		CHMs	3.76	30	6.6, 293	1–30, 3.34	[15]
	with metal ions or metal oxides	La–CS@PDA	195.3	300	3, 295	25–500, 1	[19]
		La–SBC–CS	81.54	660	4.3, 298	10–70, 2	[20]
		ZCSB	60.6	50	4, 288	5–50, 0.2	[21]
		Zr–CMC/CMCS	93.5	400	2, 298	10–100, -	[23]
		HZCSB	42.02	2160	6.7, 298	1–100, 0.75	[24]
		Ce–CS–β–CD	88.54	40	6, 303	-, 2	[27]
		Y–AlOOH@CS	45.82	30	4–6, 298	5–200, 1	[28]
		ZnCCSB	67.5	90	4–7, 295	1–1000, 1	[29]
		CB–G–Cu	53.6	720	7, 295	1–100, 1.25	[31]
		TAC@CS	43.32	40	303	20–140, 2	[32]
		Ca–CS	23.7	20	7, 298	5–50, 1	[34]
	with carbon or biochar	CS–MgCBC	221.89 *	180	3–6, 298	20–350, 2	[38]
		CS/MWCNTs	36.1	30	3, 293	25–200, 0.2	[39]
	with zeolite or mineral	La–CS–MMT	128.5	30	5.3, 303	50–300, 2	[42]
		Zr@CsKN	40.58	30	3–7, 303	80–140, -	[44]
		NCs@ZSM–5H/La	151.51	30	5, -	30–150, 0.5	[43]
	with magnetic particles	MNPsCS	1.079	720	5, 303	1–8, 30	[49]
		AFMCS	42.95	40	3–7, 313	20–140,2	[48]
		CS–Fe	15.7	-	-, 303	10–200, 10	[50]
		LC–CS–Fe	62.72	900	5, -	5–200, 2	[53]
Nanofibers		CS/Al_2_O_3_–Fe_3_O_4_	135.1	60	293	10–500, 0.5	[54]
		ZrO_2_/SiO_2_ NM	57.38	60	5, 298	1–20, -	[55]
		CS–PAA	392	60	4.5, 298	0.1–10, 2.5	[56]
Pellets and flakes		CS/flakes	6.64	40	4, -	0.5–25	[57]
		CS–EG	4.7	960	7,	10–100, 20	[58]
		CS/QCMGR	159.48	-	3–10, 303	1–100, 2	[59]
Ground and fine chitosan forms		La–BCS	16.13	40	3, 298	1–50, 0.8	[62]
		TETA–MGO/CS	353.36	50	298	1–500, -	[65]
		CS–Li@Fe_3_O_4_	98	60	2–10,	100–600, -	[67]
Nanoparticles and sponges		PFCA	7.8	360	6, 300	50, 3	[70]
		NC@GO/Zr	172.4	20	5, 313	30–150, 0.5	[72]
		LaFGO	13.12	120	3, -	5–50, 0.2	[73]

q—adsorption capacity [mg/g], t_eq(s)_—equilibrium time of adsorption process [min], T—temperature of process [K], C_P-PO4_—initial orthophosphate(V) concentration range used in Langmuir isotherm studies. * Calculated on the basis of dry filling (biochar).

**Table 2:** Effect of pH on the speciation of orthophosphate(V) forms in water.

Solution pH	Ion Order	Molecular Formula of Orthophosphate(V) Form
<2.14	-	H_3_PO_4_
2.14–7.20	I	H_2_PO_4_^−^
7.21–12.37	II	HPO_4_^2−^
>12.37	III	PO_4_^3−^

**Table 3:** Summary of substances used for the desorption of phosphates(V) from chitosan sorbents.

Desorbing Agent	Concentration	Name of Sorbent	q_m_ [mg/g]	T [K], t_eq(d)_ [min]	Number of Cycles, Desorption Efficiency [%]	Ref.
NaOH	0.1 M	Fe–CS–Alg	84.74	-, -	5, -	[17]
	3 M	La–SBC–CS	81.54	-, -	8, 94 *	[20]
	0.5 M	ZCSB	62.6	-, -	5, -	[21]
	0.5 M	HZCSB	25.58	-, -	6, 93 *	[24]
	0.1 M	Ce–C–β–CD	89	293, 60	7, -	[27]
	0.1 M	CS/MWCNTs	26.1	293, 60	5, -	[39]
	0.1 M	CS/Ca–OMMT	76	-, -	5, 94 *	[41]
	0.25 M	AFMCS	43	293, 60	8, 98 * (31 **)	[48]
	0.05 M	Fe_3_O_4_/CS/PEI	50.8	298, -	5, 90 *	[66]
NaCl	0.05 M	EP	52.1	293, 25	4, 95 *	[16]
	0.025 M	ZnCCSB	67.50	-, 30	5, -	[29]
	0.025 M	CS/QCMGR	159.48	298, 25	10, -	[59]
CuSO_4_	0.06 M	Cu–CS/Fe_3_O_4_	88.78	-, 120	5, 95 **	[81]
Mixtures	- M NaOH and	CS–La–N–20%	160	333, -	7, -	[18]
	- M NaCl					
	0.1 M NaOH and	La–CS@PDA	195.3	-, 360	5, 96 *	[19]
	0.3 M NaCl					
	1 M NaOH and	TETA–MGO/CS	353.36	-, 480	6, 60 **	[65]
	1 M NaCl					

q_m_—adsorption capacity [mg/g], t_eq(d)_—equilibrium time of desorption process [min], T—temperature of desorption [K], - no data, * first cycle result of desorption, ** last cycle result of desorption.

## Data Availability

Not applicable.

## Abbreviations

AFMCS	amine-functionalized magnetic chitosan
AgNP	silver/silver oxide nanoparticles
BC	biochar
CCM	chitosan-calcite material
Ce–CCS	Ce(III)-impregnated cross-linked chitosan complex
Ce–CS–β-CD	chitosan-β-cyclodextrin sorbent with embedded cerium Ce^3+^ ions
CS/Ca–OMMT	chitosan/Ca-organically modified montmorillonite
CS/MWCNTs	chitosan modified with multi-walled carbon nanotubes
CSB	chitosan bead
CSB–Cu	chitosan bead (CSB) modified copper ion (Cu(II))
CSBent	chitosan supported bentonite
CSB–G–Cu	chitosan bead (CSB) modified copper ion (Cu(II) glutaraldehyde cross-linked
CS–Fe	Fe(III)-doped chitosan composite
CS–Fe–CL	cross-linked Fe(III)-chitosan composite
CSH	chitosan hydrogel
CSHB	chitosan hydrogel beads (non-modified)
CSHs	chitosan hydrogel granules (non-modified)
CSHs-ECH	chitosan hydrogel granules cross-linked with epichlorohydrin
CS–La–N–20%	hydrogel immobilized with La(OH)_3_ nanoparticles within a quaternary–aminated chitosan
CS–Li@Fe_3_O_4_	magnetic Fe_3_O_4_ doped lignin-chitosan-based microspheres
CS–MgCBC	chitosan modified magnesium impregnated corn straw biochar and glutaraldehyde cross-linked
CSNP	chitosan nanoparticles
Cu(II)	loaded CS chitosan beads directly after the saturated adsorption of copper(II) ions
CZD	chitosan pre-modified diatomite composited with ZIF–8
EP	epichlorohydrin
Fe_3_O_4_/CS/PEI	polyethylenimine (PEI)-grafted chitosan (CS) core–shell
Fe–CS–Alg	Fe^3+^ loaded chitosan and alginate biopolymeric hybrid beads
FTIR	Fourier transform infrared spectroscopy analysis
GA	glutaraldehyde
GO	graphene oxide
HZCSB	hydrogel beads composed of zirconia and chitosan
i–PO_4_	phosphate(V) ions (PO_4_^3−^, HPO_4_^2−^, H_2_PO_4_^−^)
La–BCS	lanthanum/chitosan-modified bentonite
La–CS@PDA	lanthanum-modified chitosan with a protective coating of polydopamine
La–CTS–0X	non-cross-linked lanthanum-chitosan
La–CTS–1X/2X	glutaraldehyde cross-linked lanthanum-chitosan composites
LaFGO	lanthanum-flocculated graphene oxide
La–SBC–CS	lanthanum-modified sludge biochar chitosan
LC–CS–Fe	chitosan modified with acid-leached carbon waste and FeCl_3_
MCS	magnetic chitosan composite
MGO	magnetic graphene oxide
MGO/CS	magnetic graphene oxide chitosan composite
MK-CS	chitosan-encapsulated magnetic kaolin beads
MNP	magnetic nanoparticles
MNPsCS	chitosan composite of magnetic nanoparticles dispersed
nano–ZnO–CS	sorbents developed from CS and zinc compounds
NC@GO/Zr	Zr-nanochitosan/graphene oxide composite
PDA	polydopamine
PEG	polyethylene glycol
PEI	polyethylenimine
PFC	polyurethane foam impregnated with chitosan nanoparticles
PFCA	polyurethane foam impregnated with chitosan nanoparticles and silver/silver oxide nanoparticles
PUF	polyurethane foam
SEM	scanning electron microscope
SF	sponge filter
SPE	solid phase extraction
TAC	tetra-amine copper(II)
TAC@CS	composite chitosan beads by grafting tetra-amine copper(II) (TAC)
TETA	triethylene tetramine
TETA–MGO/CS	triethylene tetramine-functionalized magnetic graphene oxide chitosan composite
T–P	total phosphorous
ZCSB	chitosan hydrogel beads modified with Zr^4+^ ions
Zn(II)–CS	sorbents developed from CS and zinc compounds
ZnCCSB	chitosan beads carboxylated cross-linked with glutaraldehyde loaded with Zn(II)
ZnO–CS	sorbents developed from CS and zinc compounds
Zr–CMC/CMCS	Zr(IV)-cross-linked carboxymethyl cellulose/carboxymethyl chitosan hydrogel
ZrO_2_/SiO_2_ NM	nanofibrous membranes modified with ZrO_2_ and SiO_2_ nanoparticles
